# Supplementary material for: The Mitogen-Activated Protein Kinase PlMAPK2 Is Involved in Zoosporogenesis and Pathogenicity of Peronophythora litchii
Source: Int J Mol Sci. 2021 Mar 29;22(7):3524. doi: 10.3390/ijms22073524 (PMC8036616; doi:10.3390/ijms22073524)
Supplement: Supplementary file 1 [file ijms-22-03524-s001.zip › ijms-1110580 suppl revised/Figure S1-S3.pdf]

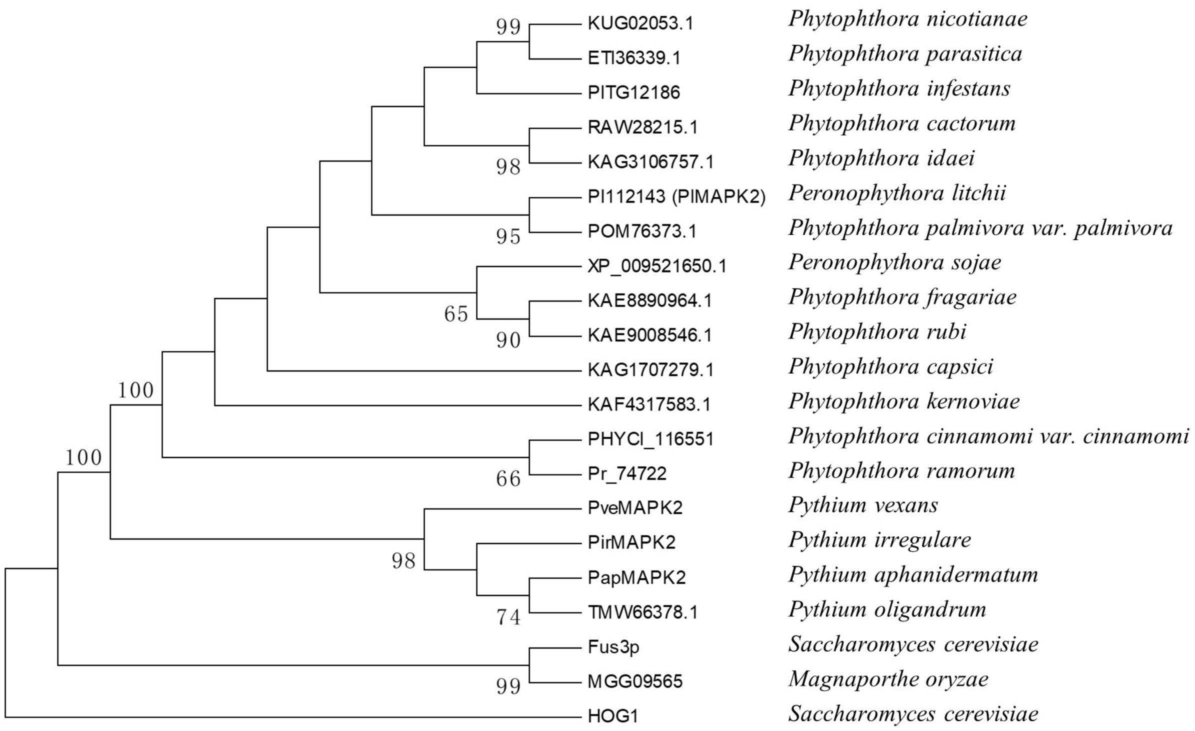

Figure S1. Phylogenetic analysis of putative PlMAPK2 orthologs based on the amino acid sequences (Table S2). The neighbor-joining tree was constructed by MEGA 6.0 using the neighbor-joining algorithms with 1000 bootstrap replications [23]. Bootstrap values greater than 50 were displayed.

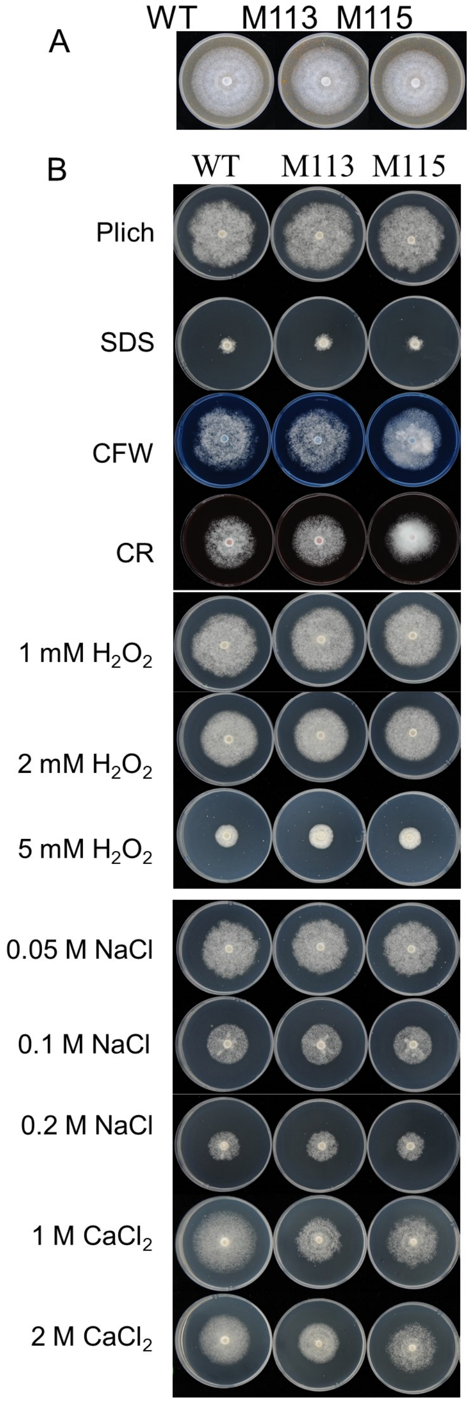

Figure 2. PlMAPK2 is not associated with growth and responses to various stresses. (**A**) WT and *PlMAPK2* mutants were inoculated on CJA medium, photographs were taken 5 days after inoculation. (**B**) WT and *PlMAPK2* mutants were inoculated on Plich medium with or without indicated chemicals (350 µg/mL CFW, 25 µg/mL SDS, 100 µg/mL CR, 1 mM H_2O_2, 2 mM H_2O_2, 5 mM H_2O_2 0.05 mM NaCl, 0.1 mM NaCl, 0.2 mM NaCl, 0.1 mM $CaCl_2$ and 0.2 mM $CaCl_2$.). Photographs were taken 5 days after inoculation.

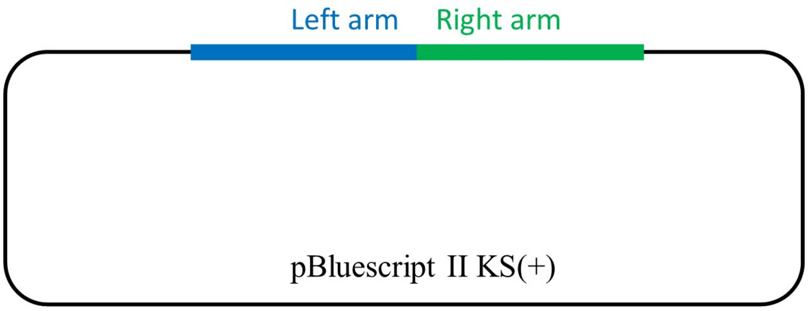

Figure 3. Structure of pBluescript II KS(+)::MAPK2. Left arm and right arm were inserted in pBluescript II KS(+) plasmid.
